# Preparation of Y_2_O_3_/TiO_2_-Loaded Polyester Fabric and Its Photocatalytic Properties under Visible Light Irradiation

**DOI:** 10.3390/polym14142760

**Published:** 2022-07-06

**Authors:** Yu Ren, Ziyao Zhao, Wenwen Jiang, Guangyu Zhang, Yuxin Tan, Yongyin Guan, Long Zhou, Li Cui, Sung Woong Choi, Mei-Xian Li

**Affiliations:** 1 School of Textile and Clothing, Nantong University, Nantong 226019, China; ren.y@ntu.edu.cn (Y.R.); 2112310010@stmail.ntu.edu.cn (Z.Z.); jiangwenwenntu@163.com (W.J.); zgyu85@ntu.edu.cn (G.Z.); tanyuxin19@163.com (Y.T.); 2 Xin Feng Ming Group, Huzhou Zhongshi Technology Co., Ltd., Huzhou 313000, China; gyy@xfmgroup.com (Y.G.); zl@xfmgroup.com (L.Z.); cl@xfmgroup.com (L.C.); 3 Department of Mechanical System Engineering, Gyeongsang National University, Tongyeong 53064, Korea; younhulje@gmail.com

**Keywords:** Y_2_O_3_, TiO_2_, polyester, composite photocatalysis, dye degradation, visible light

## Abstract

In this study, Y_2_O_3_/TiO_2_-loaded polyester fabric was prepared to improve the catalytic activity of the TiO_2_ and to increase its reuse efficiency. The samples were systematically characterized by scanning electron microscopy (SEM), X-ray diffractometry (XRD), X-ray photoelectron spectroscopy (XPS), and infrared spectroscopy (FT-IR). Furthermore, the degradation performance of methyl orange in the presence of simulated visible light irradiation was also investigated. The results showed that the TiO_2_ in the Y_2_O_3_/TiO_2_ composite photocatalyst was suitably anatase. In addition, Y_2_O_3_/TiO_2_-loaded polyester fabric had higher photocatalytic performance than that of pure polyester fabric under visible light and the degradation rate reached 83% after 120 min of light exposure but remained above 50% after repeated exposure (three times). Compared to the pure polyester fabric, Y_2_O_3_/TiO_2_-loaded polyester fabric had self-cleaning effects in methyl blue and soy sauce solutions under visible light.

## 1. Introduction

Recently, the development of functional and intelligent textiles has attracted considerable attention worldwide [1] and among these, fabrics with photocatalytic and self-cleaning properties have been widely studied [2,3]. The energy band structure of semiconductors contributes to the theoretical basis of photocatalytic reactions [4], and most semiconductor photocatalysts are of the n-type semiconductor material with a band gap between the valence and conduction bands. An incident photon makes the electrons in the valence band become excited and transition to the conduction band after energy absorption, leaving holes in the valence band. At the same time, many photo-generated holes (h^+^) and electrons (e^−^) will be produced in the conduction band and valence band, which makes the valence band reduced, and the electrons in the conduction band oxidized, leading to oxidation or reduction of the contaminants. The surface of the fabric is modified by loading a photocatalyst [5], which will generate electrons and holes with redox ability under sunlight to purify pollutants [6]. 

Commonly used photocatalysts include TiO_2_ [7,8], SnO_2_ [9,10], ZnO [11,12], and ZrO_2_ [13,14]. Among them, TiO_2_, as a photosensitive semiconductor, has attracted widespread attention due to its high stability and non-toxicity [15] and therefore, has been widely used in the field of photocatalysis. However, the wide band gap of TiO_2_ (3.2 eV) means that it can only be photo-activated under ultraviolet light, resulting in low collection efficiency of the visible light in sunlight [16]. It has been reported that TiO_2_ has various size, nanostructure, and polymorphs (anatase polymorph, rutile polymorph, mixed anatase-rutile polymorphs, etc.) [17]. Moreover, the TiO_2_ lattice is distorted by doping with rare earth oxides, which can accept more light to excite holes and generate stronger surface free radicals causing oxidation of the pollutant molecules [18]. In addition, rare earth elements have a special electronic structure of 4fx5dy, which plays an important role in the photo-generated charge transfer between the 4f energy level and the conduction band or valence band of TiO_2_ [19]. As a heavy rare earth oxide, Y_2_O_3_ has a relatively small atomic radius with the energy gap of 6.3 eV [20], which can improve the photocatalytic activity of TiO_2_ [21] and it has been found that an appropriate combination of Y_2_O_3_ can effectively improve the photocatalytic activity of TiO_2_ in the visible light band [22].

To date, Jiang et al. [23] prepared Ag-loaded TiO_2_/Y_2_O_3_ hollow microspheres with a double shell structure of melamine formaldehyde using the sol-gel method and high temperature calcination reduction. The results showed that the degradation efficiency of methyl orange by TiO_2_/Ag/Y_2_O_3_ increased seven times under visible light when compared to TiO_2_ hollow spheres. Using the combustion and precipitation method, Li et al. [24] synthesized a novel composite photocatalyst TiO_2_/Y_2_O_3_:Yb^3+^, Tm^3+^ and the photocatalytic activity of this composite was higher than that of pure TiO_2_ under solar light irradiation. Ahmed et al. [25] prepared an Eu-doped Y_2_O_3_:TiO_2_ nanocomposite via the sol-gel synthesis process and the optical properties of the synthesized nanocomposite were investigated at different temperatures, and its application as a potential material for thermographic phosphor and in LED fabrication was investigated.

Although photocatalysts from the above research exhibited enhanced photoactivity, photocatalysts mostly exist in the form of nano powders, which are difficult to separate and recover from bodies containing water causing a waste of resources. Therefore, the development of matrix materials loaded with photocatalysts to improve the service efficiency of photocatalysts is urgently needed. Zhu et al. [26] developed a nanoparticle/polymeric capsule via an in situ growth method, of which lead-free bismuth halide peroskite nanocrystals and covalent organic frameworks are used as core and shell, respectively, demonstrating good water processability as well as excellent photocatalytic performance. Polyester fabrics are also widely used because of their low cost, good durability, and high stability [27,28]. However, polyester fabric macromolecules lack polar functional groups on their surfaces as well as a highly crystalline structure. It is reported that nanoparticle/polymer composites designed with combination of initial interpenetrating polymer network structure and organo-inorganic interpenetrating polymer network structure could significantly influence the relaxation behavior of the composites [29]. Moreover, the ordered polymer chains in the crystal lattice are too dense, which makes low bonding strengths when combining them with inorganic nano-photocatalytic materials [30]. Polar functional groups can be introduced onto the surface by chemical modification and plasma treatment to improve the speed of bonding of the inorganic powder and fabric surface [31].

In this study, tetrabutyl titanate and yttrium nitrate hexahydrate were used as raw materials to prepare Y_2_O_3_/TiO_2_ with photocatalytic activity in the visible light range using the sol-gel method. Y_2_O_3_/TiO_2_ was modified by 3-aminopropyltrimethoxysilane (APTMS) to enhance, the interaction between Y_2_O_3_/TiO_2_ and polyester fabrics through Si-O-Ti bonds [27] for the preparation of Y_2_O_3_/TiO_2_ loaded polyester fabrics. The samples were systematically characterized by scanning electron microscopy (SEM), X-ray diffractometry (XRD), X-ray photoelectron spectroscopy (XPS), and infrared spectroscopy (FT-IR). Furthermore, methyl orange was used as an organic pollutant and the photocatalytic self-cleaning properties of modified polyester fabrics were studied under xenon lamp irradiation.

## 2. Materials and Methods

### 2.1. Materials

Polyester fabrics were provided from the Jiangsu Kuangda group, and the reagents used in this study, namely tetrabutyl titanate (Jiangsu Qiangsheng Functional Chemical Co., Ltd. Suzhou, China), anhydrous alcohol (Runjie Chemical Reagent Co., Ltd., Shanghai, China), glacial acetic acid (Hubao Chemical Reagent Co., Ltd., Yangzhou, China), yttrium nitrate hexahydrate (Aladdin Biochemical Technology Co., Ltd., Shanghai, China), methyl orange (Chengji Chemical Trading Co., Ltd., Nantong, China), and methyl blue (Chengji Chemical Trading Co., Ltd., Nantong, China), were all of analytical grade. 

### 2.2. Preparation of Y_2_O_3_/TiO_2_

**Figure 1:**
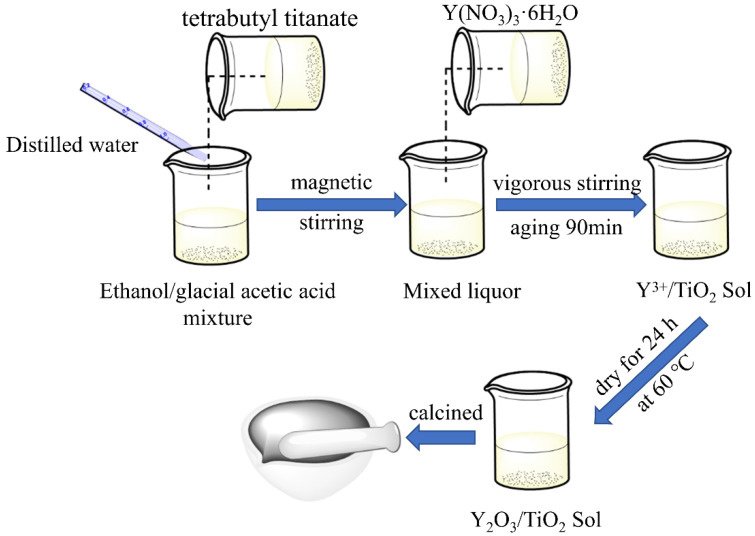
Schematic diagram of the Y_2_O_3_/TiO_2_ preparation process.

First, 46 mL of absolute ethanol and 6 mL of glacial acetic acid were mixed followed by the addition of 10 mL of tetrabutyl titanate under vigorous stirring, and then 3 mL of distilled water and an appropriate amount of Y(NO_3_)_3_⋅6H_2_O were sequentially added with vigorous stirring for 60 min. After this, the xerogel was prepared via aging for 90 min and drying at 60 °C for 24 h. Finally, the xerogel was calcined in a resistance furnace at 500 °C for 2 h to obtain a Y_2_O_3_/TiO_2_ catalyst. Composite catalysts containing different mole fractions (0.5%, 0.75%, 1%) of Y_2_O_3_ were prepared with different molar ratios of Y(NO_3_)_3_⋅6H_2_O. The preparation flow chart of Y_2_O_3_/TiO_2_ is shown in Figure 1.

### 2.3. Preparation of Y_2_O_3_/TiO_2_-Loaded Polyester Fabrics

First, 0.5 g of Y_2_O_3_/TiO_2_ was dispersed in 100 mL of diluted ethanol solution (V_ethanol_:V_distilled water_ = 95:5), and then 5 mL of 3-aminopropyltrimethoxysilane (APTMS) was added and sonicated for 30 min. The mixture was then centrifuged and washed with alcohol, followed by drying in a vacuum oven at 70 °C to obtain amino functionalized Y_2_O_3_/TiO_2_, which was abbreviated to NH_2_-Y_2_O_3_/TiO_2_. The polyester fabrics were immersed in a solution containing 1 mg/L NH_2_-Y_2_O_3_/TiO_2_, and then oscillated for 1 h to ensure that ester groups in polyester fabrics and amino groups in NH_2_-Y_2_O_3_/TiO_2_ were fully reacted, followed by drying at 60 °C to obtain Y_2_O_3_/TiO_2_ loaded polyester fabrics.

**Figure 2:**
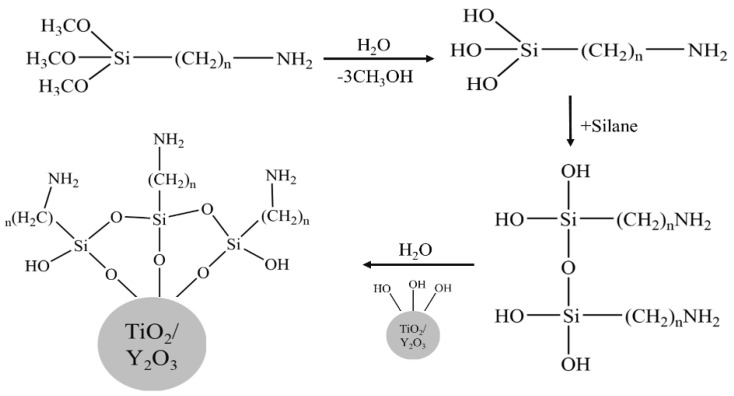
Reaction scheme for the surface functionalization of Y_2_O_3_/TiO_2_ by APTMS.

**Figure 3:**
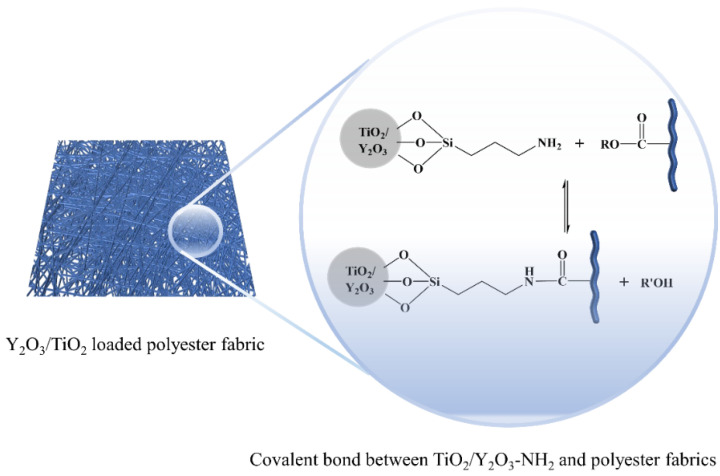
Schematic illustration of the interaction between Y_2_O_3_/TiO_2_-NH_2_ and polyester fabrics.

The schematic diagram of APTMS modified Y_2_O_3_/TiO_2_ is shown in Figure 2 and the local enlarged view of Y_2_O_3_/TiO_2_-NH_2_ nanoparticles bonded on polyester fabrics is shown in Figure 3.

### 2.4. Photocatalytic Degradation of Methyl Orange

Methyl orange solution with a concentration of 20 mg/L was prepared, and then 100 mL of the prepared solution taken to the photocatalytic materials, followed by magnetically stirring for 30 min in the dark to reach the adsorption equilibrium. A xenon light source (300 W) was used to simulate a visible light source to analyze the visible light catalytic performance of the photocatalyst. The supernatant was extracted at regular intervals and the absorbance at a wavelength of 464 nm was measured by a dual-beam UV–Vis spectrophotometer (TU-1900).

### 2.5. Surface Morphology Analysis

The surface characteristics and crystal shape of the catalysts were analyzed by using ZEISS Gemini SEM 300 field emission scanning electron microscope.

### 2.6. Crystal Structure Analysis

The photoelectric spectrum was analyzed using X-ray photoelectron spectroscopy with the model K-Alpha+ produced by Thermo Scientific (Waltham, MA, USA).

### 2.7. Chemical Composition Analysis

The phase composition of the catalyst was analyzed by using the D/MAX-2500 X-ray diffractometer (XRD) produced by Rigaku Corporation (Tokyo, Japan), with a 40 kV working voltage, 100 mA current as well as a 10°–80° scanning range (2θ).

The samples were irradiated with infrared light in the scanning range of 8300–350 cm^−1^ using a model of Spectrum Two equipment produced by Perkin Elmer (Boston, MA, USA). When the vibration of the molecular groups was the same as the frequency of the infrared spectrum, they could be distinguished. This group was compared with the standard infrared spectrum to determine the composition of the sample.

### 2.8. UV-Vis Analysis

A UH4150 UV–Vis spectrophotometer (UV–vis), produced by Hitachi (Tokyo, Japan) was used to measure the UV–visible light absorption spectrum of the samples at a scanning speed of 1200 nm/min at intervals of 1 nm.

### 2.9. Self-Cleaning Property

To investigate the self-cleaning performance of the pure polyester fabric and Y_2_O_3_/TiO_2_-loaded polyester fabric under simulated visible light, 0.1 mL of methyl blue solution and soy sauce were dropped on the polyester fabric and that loaded with Y_2_O_3_/TiO_2_ and irradiated under a xenon lamp for 18 h and sample images taken every 6 h.

## 3. Results and Discussion

### 3.1. Surface Morphology Analysis

**Figure 4:**
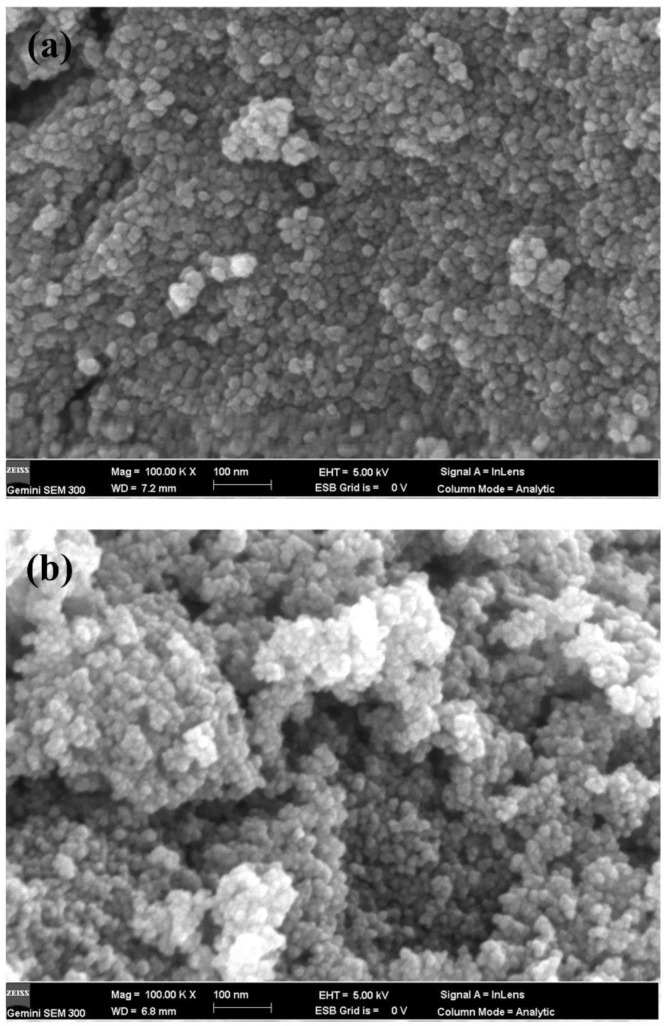
Scanning electron micrograph images of photocatalyst (**a**) TiO_2_ (×100,000), (**b**) Y_2_O_3_/TiO_2_ (×100,000).

Figure 4 shows scanning electron microscope images of TiO_2_ and Y_2_O_3_ (the molar content is 0.75%) under 100,000 times magnification. It can be seen from Figure 4a that the prepared TiO_2_ nanoparticles were spherical, with a diameter of about 10 nm, and the distribution is relatively uniform. As shown in Figure 4b, Y_2_O_3_/TiO_2_ remained spherical without differences in morphology due to the low content of Y_2_O_3_. However, there were slight clusters between the particles because the diameters of the particles were slightly reduced after recombination, and the nanoparticles with small diameters were more likely to agglomerate. This showed that addition of an appropriate amount of Y_2_O_3_ inhibited the growth of TiO_2_ grains.

**Figure 5:**
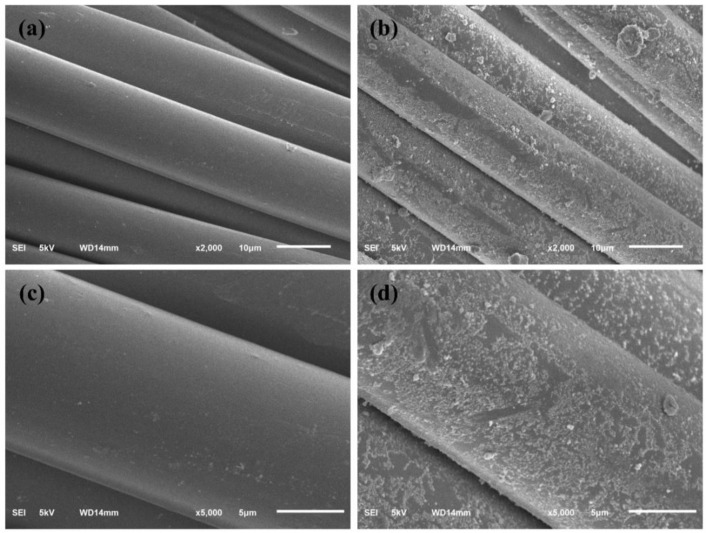
Scanning electron micrograph of photocatalytic materials (**a**) polyester fabric (×2000), (**b**) Y_2_O_3_/TiO_2_ loaded polyester fabric (×2000), (**c**) polyester fabric (×5000), (**d**) Y_2_O_3_/TiO_2_-loaded polyester fabric (×5000).

Figure 5 shows scanning electron photomicrographs of pure polyester fabric loaded with Y_2_O_3_/TiO_2_ (the molar content of Y_2_O_3_ was 0.75%) under two different magnifications of 2000× and 5000× Figure 5a,c represent surface morphology of pure polyester fabric under 2000× and 5000× magnification, respectively. From the two images, it can be observed that the surface of the pure polyester fabrics was flat and smooth. Figure 5b,d represent surface morphology of polyester fabrics loaded with amino functionalized Y_2_O_3_/TiO_2_ composite photocatalyst under 2000× and 5000× magnification. From Figure 5b, it can be observed that the polyester fabric was covered with white particles resulting in a rough surface. Under a magnification of 5000×, it could be observed that white particles of Y_2_O_3_/TiO_2_ were distributed evenly and tightly on the surface of the fabric. It showed that the prepared Y_2_O_3_/TiO_2_ catalyst was successfully loaded onto the polyester fabric and evenly distributed on the surface of the polyester, which played an important role in photocatalytic performance under visible light.

### 3.2. Crystal Structure Analysis

**Figure 6:**
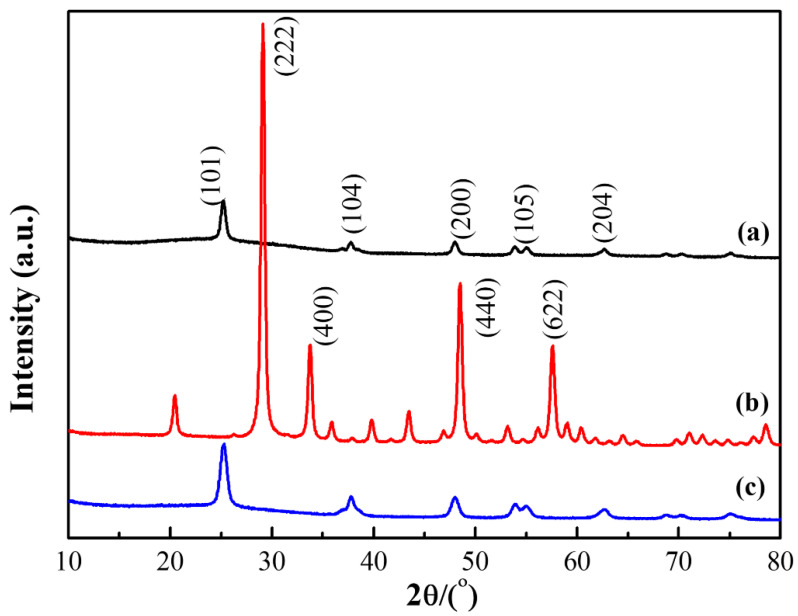
X-ray diffraction pattern of the photocatalyst (**a**) TiO_2_, (**b**) Y_2_O_3_, and (**c**) Y_2_O_3_/TiO_2_.

The crystalline structure of the three photocatalytic materials, namely TiO_2_, Y_2_O_3_, and Y_2_O_3_/TiO_2_ (the molar content of Y_2_O_3_ is 0.75%) are shown in Figure 6. As shown in Figure 6a, the diffraction peaks appeared at 2θ of 25.26°, 37.2°, 48°, 54.04°, and 62.84°, which corresponded to the crystal plane indices of (101), (104), (200), (105), and (204) of the TiO_2_ anatase phase, respectively [32]. In addition, contrast diffraction peak positions were in accordance with TiO_2_ standard diffraction card (JCP-DS84-1285), indicating that the prepared TiO_2_ was anatase type [33]. In Figure 6b, the diffraction peaks at 29.1°, 33.79°, 48.54°, and 57.62° corresponded to the (222), (400), (440), and (622) planes of Y_2_O_3_, respectively. The positions of the diffraction peaks are also consistent with the Y_2_O_3_ standard diffraction card (JCP-DS25-1200), showing good crystallization and crystal integrity [34]. However, the diffraction peak of Y_2_O_3_ could not be seen in Figure 6c; this may be due to the low content of Y_2_O_3_ which may not have been enough to change the crystal form of TiO_2_ [35], where TiO_2_ remained the titanite type in the Y_2_O_3_ composite photocatalyst [36].

**Figure 7:**
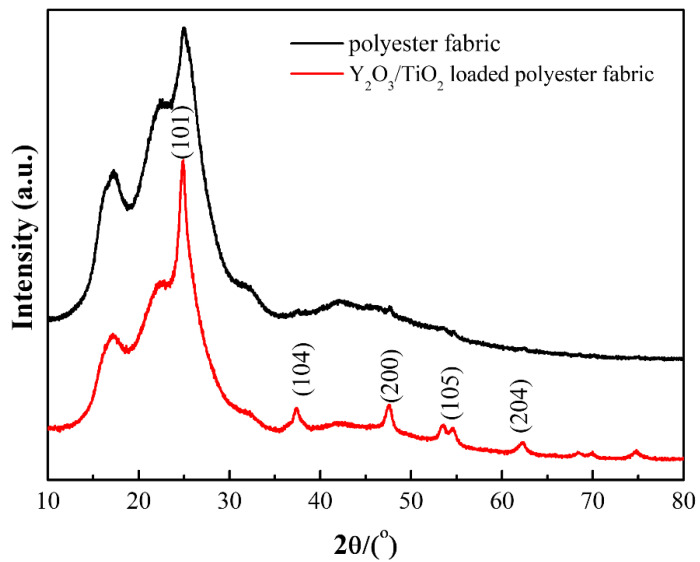
X-ray diffraction patterns of polyester fabric and Y_2_O_3_/TiO_2_-loaded polyester fabric.

The crystalline structures of the polyester fabric and Y_2_O_3_/TiO_2_-loaded polyester fabric (the molar content of Y_2_O_3_ is 0.75%) are shown in Figure 7. The diffraction peaks at 17.2°, 22.8°, and 25.7° were the characteristic peaks of polyester fabric [37], while those at 25.5°, 37.2°, 48°, 54.04°, and 62.84° corresponded to the crystal plane indices of (101), (104), (200), (105), and (204) of the TiO_2_ anatase phase [33], respectively. The characteristic peak of TiO_2_ at 25.5° was coincident with that of polyester. Furthermore, all the other diffraction peaks coincided with the characteristic peaks of the Y_2_O_3_/TiO_2_ composite catalyst in Figure 7, which indicated that the Y_2_O_3_/TiO_2_ composite catalyst was successfully loaded on the polyester fabric.

### 3.3. Chemical Composition Analysis

**Figure 8:**
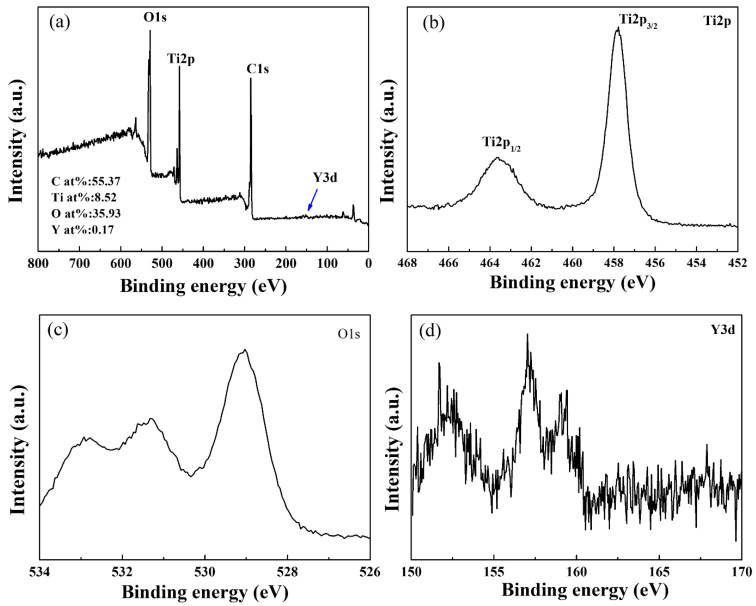
XPS survey of Y_2_O_3_/TiO_2_-loaded polyester fabric: (**a**) full peak of Y_2_O_3_/TiO_2_-loaded polyester fabric, (**b**) Ti2p peak, (**c**) O1s peak, (**d**) Y3d peak.

Figure 8 represents the XPS full spectrum of the Y_2_O_3_/TiO_2_-loaded polyester fabric (the molar content of Y_2_O_3_ is 0.75%). Figure 8b–d are the high resolution XPS image of Ti, O, and Y in the Y_2_O_3_/TiO_2_-loaded polyester fabric. It can be seen from Figure 8a that Y_2_O_3_/TiO_2_-loaded polyester fabric mainly contained Ti, Y, O, and C elements, and the C peak and O peak were mainly from polyester [38], while the Ti peak, O peak, and Y peak were from Y_2_O_3_/TiO_2_ composite photocatalyst in polyester fabric. In Figure 8b, two Ti2p peaks (Ti2p3/2 and Ti2p1/2) were detected at 456.05 eV and 463 eV, respectively, indicating the presence of Ti^4+^ on the polyester fabric [37,39]. Figure 8c is the XPS spectrum of the O1s region of Y_2_O_3_/TiO_2_-loaded polyester fabric, which is mainly divided into three peaks. The O element appearing at 529.03 eV shows the Ti-O-Y bond, while that at 529.9 eV indicates O^2−^ in the sample [40]. The content of O^2−^ was relatively high, indicating that O in the Y_2_O_3_/TiO_2_ composite photocatalyst mainly existed in the form of the negative divalent [41]. Moreover, the O element at 532 eV was derived from the -OH produced by the adsorption and dissociation on the sample surface [39], indicating that the holes generated by the Y_2_O_3_/TiO_2_ composite photocatalyst after light excitation reacted with the adsorbed H_2_O or OH ions on the surface, forming the hydroxyl radicals, which helped to improve the photocatalytic performance of Y_2_O_3_/TiO_2_ composite photocatalyst [42].

**Figure 9:**
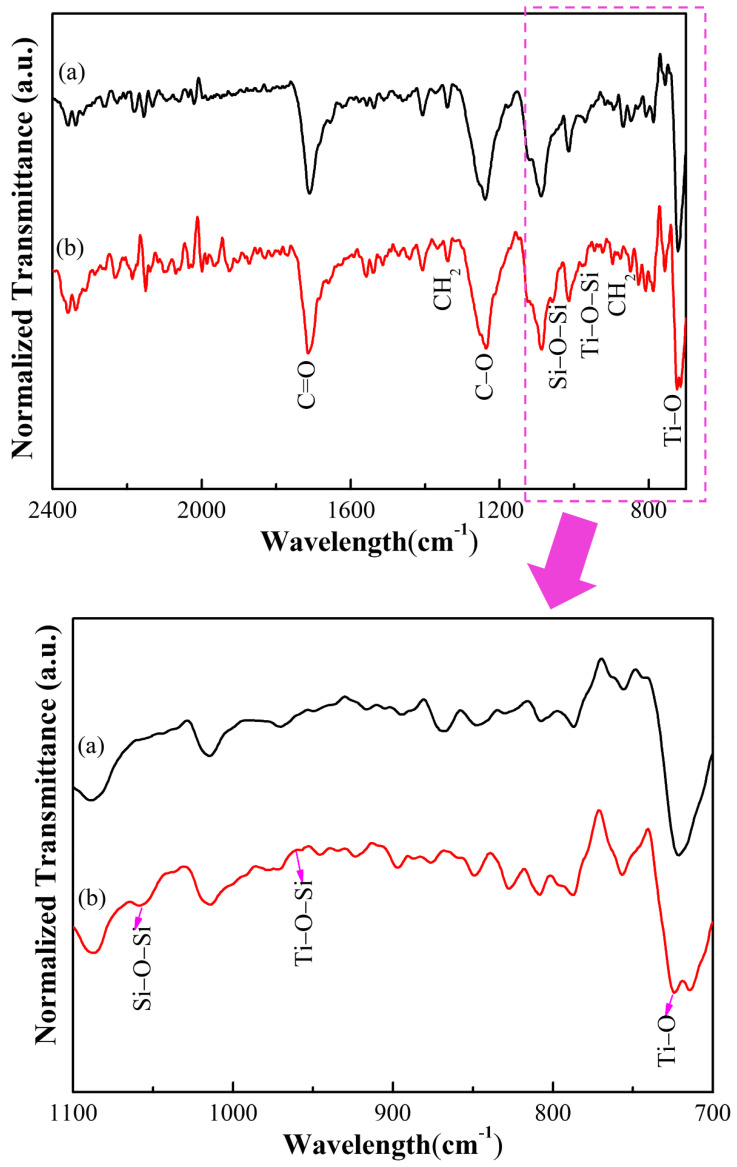
Infrared spectra of photocatalytic materials (a) polyester fabric, (b) Y_2_O_3_/TiO_2_-loaded polyester fabric.

Figure 9 shows the Fourier infrared spectra of two photocatalytic materials, the polyester fabric, and Y_2_O_3_/TiO_2_-loaded polyester fabric (the molar content of Y_2_O_3_ is 0.75%). As shown in Figure 9, the polyester fabric had an obvious characteristic peak at 1714.47 cm^−1^, which was the peak of polyester C=O stretching vibration. Moreover, peaks at 1339.37 cm^−1^ and 1240.05 cm^−1^ were attributed to CH_2_ groups and CO stretching vibration, respectively [43]. In addition, the peaks at 1058.77 cm^−1^ and 1014.41 cm^−1^ corresponded to Si-O-Si stretching vibration and the trans conformation band of the CH bond on the benzene ring [44], respectively. Furthermore, those at 960.71 cm^−1^ and 850.48 cm^−1^ were assigned to Ti-O-Si bonds and CH_2_ in-plane rocking vibration, respectively. The peak of Ti-O bond stretching vibration and the absorption peak of CH_2_ out-of-plane bending on the benzene ring of the polyester fabric overlapped at 724.17 cm^−1^. This indicated that the Y_2_O_3_/TiO_2_ was modified by 3-aminopropyltrimethoxysilane (APTMS), in which the effect of organosilicon on the adsorption and interaction of Y_2_O_3_/TiO_2_ was attained through the formation of Si-O-Ti bonds. During this, the Y_2_O_3_/TiO_2_ was loaded onto the surface of the polyester fabric, where the silanized Y_2_O_3_/TiO_2_ was nucleophilically attacked by the introduction of amine groups, causing breakage of the ester bonds and the formation of the amide bonds to form a covalent bond with the surface of the polyester fabric. Eventually, Y_2_O_3_/TiO_2_ after amino functionalization treatment was successfully loaded on the polyester fabric.

### 3.4. UV–Vis Analysis

**Figure 10:**
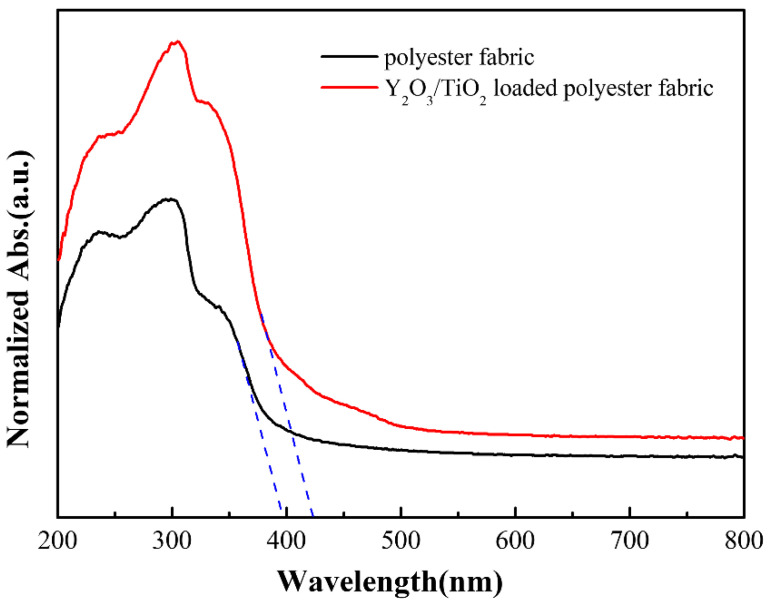
UV–Visible absorption spectrum of the photocatalytic materials.

Figure 10 shows the UV–Vis absorption spectra for the polyester fabric and Y_2_O_3_/TiO_2_-loaded polyester fabric (the molar content of Y_2_O_3_ is 0.75%). When compared to the polyester fabric, the Y_2_O_3_/TiO_2_-loaded polyester fabric not only absorbed in the entire spectrum, but also was enhanced in the entire visible range. In addition, the absorption spectrum of Y_2_O_3_/TiO_2_-loaded polyester fabric appeared red shift at the boundary of the visible range due to changes in the conduction band and valence band of the Y_2_O_3_/TiO_2_ photocatalysts [19]. As shown in Figure 10, the absorption edge of the polyester fabrics and the Y_2_O_3_/TiO_2_-loaded polyester fabrics was about 390 nm and 420 nm, respectively. According to the formula Eg = 1240/λ, the corresponding band gaps were calculated as 3.17 eV and 2.95 eV and compared to polyester fabric, the band gap of Y_2_O_3_/TiO_2_-loaded polyester fabric was narrower, indicating a higher light absorption rate to improve the photocatalytic performance of the fabric.

### 3.5. Degradability Analysis

**Figure 11:**
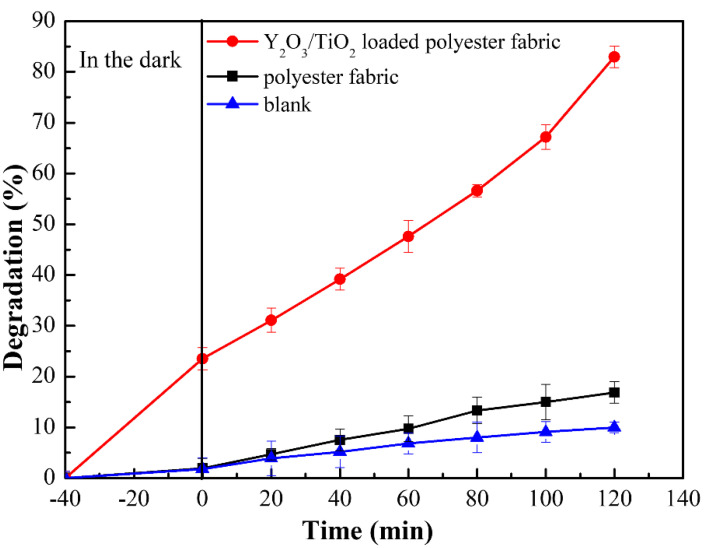
Degradation of methyl orange under xenon lamp irradiation.

Figure 11 shows the degradation curves with methyl orange solutions under irradiation with a 300 W xenon lamp for 120 min for the polyester fabric and Y_2_O_3_/TiO_2_-loaded polyester fabric (with a molar content of 0.75% of Y_2_O_3_). When samples were left in the dark for 40 min, some of the methyl orange solution could be absorbed by the samples and reach adsorption equilibrium of the catalyst and the solution, then the methyl orange solution was irradiated with different fabrics using the 300 W xenon lamp to simulate sunlight for 120 min. The supernatant was taken every 20 min for absorbance testing, and the control samples were 100 mL and 20 mg/L of methyl orange solution. As shown in Figure 11, the methyl orange solution hardly degraded in the absence of fabrics. However, the methyl orange solution added to the polyester fabric was slightly degraded under irradiation, whereas that added to Y_2_O_3_/TiO_2_-loaded polyester fabric was degraded at a rapid rate. After 120 min of light exposure, the removal efficiency of the polyester fabric, Y_2_O_3_/TiO_2_-loaded polyester fabric and methyl orange were 16.9%, 83%, and 10%, respectively.

**Figure 12:**
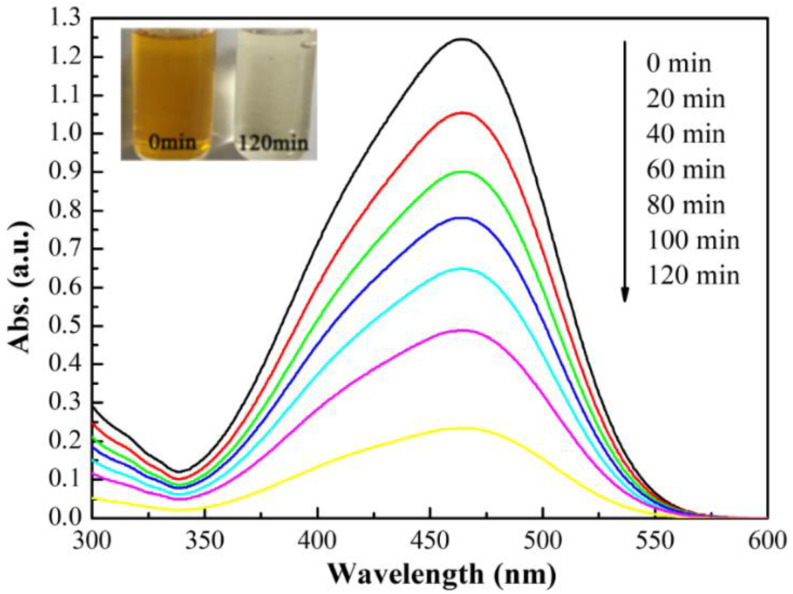
Absorbance changes in methyl orange (20 mg/L) on the Y_2_O_3_/TiO_2_-loaded polyester fabric.

From Figure 12, it can be seen that methyl orange had a maximum absorption at 464 nm [45], and the absorbance was the highest at 0 min whereas it was the smallest after 120 min of irradiation with the xenon lamp. Furthermore, the color of the methyl orange solution changed significantly from bright orange (0 min) to colorless (120 min), indicating the decrease of the chromophore and the destruction of the benzene ring of methyl orange under the irradiation of the xenon lamp [46]. In addition, no new peaks appeared during the whole irradiation process, indicating that the methyl orange was successfully degraded.

**Figure 13:**
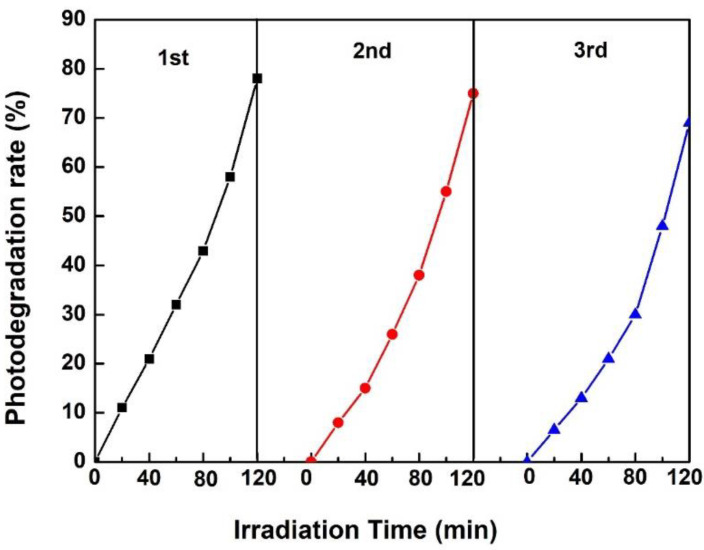
Cycles of methyl orange (20 mg/L) by Y_2_O_3_/TiO_2_-loaded polyester fabric.

The used polyester fabric loaded with Y_2_O_3_/TiO_2_ (the molar content of Y_2_O_3_ is 0.75%) was recycled, collected, washed, and dried. Then, the photocatalytic degradation of methyl orange on the dried polyester fabric loaded with Y_2_O_3_/TiO_2_ was carried out three times under visible light irradiation. Figure 13 shows the cycles for the degradation of methyl orange by Y_2_O_3_/TiO_2_-loaded polyester fabric under visible light. The degradation efficiency changes from 80% to 70%. This result indicates that the photocatalytic activity does not obviously deteriorate after three recycles for the photodegradation of methyl orange, revealing the good stability of Y_2_O_3_/TiO_2_-loaded polyester fabric.

### 3.6. Photocatalytic Reaction Kinetics

Several recent reports claim that Langmuir–Hinshelwood kinetic model can describe the photocatalytic degradation of heterogeneous TiO_2_ well [47,48].

The form of the photocatalytic degradation rate model can be expressed by the following equation [49,50].

(1) $$r=-\frac{dC}{dt}=\frac{k_{r}k_{s}C_{0}}{1+k_{s}C_{0}}$$

where *C*_0_ is the organic concentration, *K_r_* is reaction rate constant, *K_s_* is the apparent adsorption constant, and *t* is the time of reaction. The term *K_s_C*_0_ is often negligible when the concentration is low, and the reaction rate can be expressed as pseudo-first-order model as follows:

(2) $$-\frac{dC}{dt}=k_{r}K_{s}C_{0}=K_{app}t$$

where *K_app_* is the apparent first-order reaction rate.

Integration of the equation yields the following equation:

(3) $$\ln\left(\frac{C}{C_{0}}\right)=-K_{app}t$$

**Figure 14:**
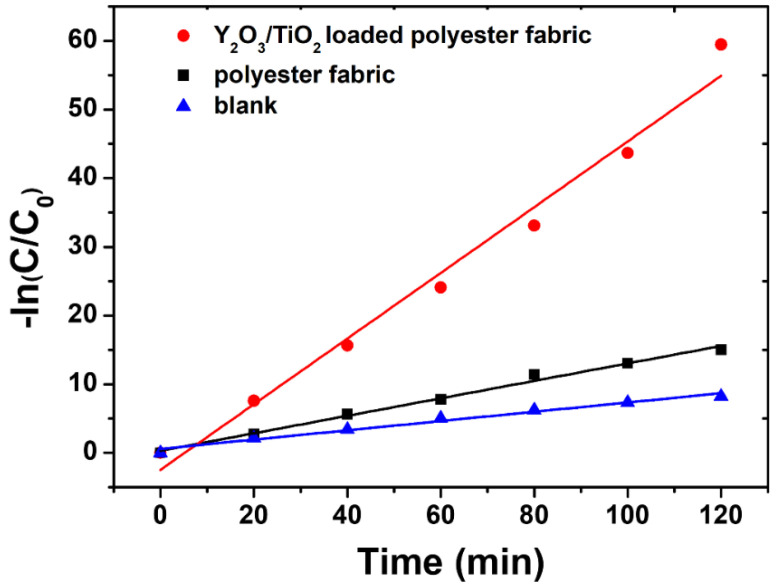
First-order kinetic plot of −ln(*C/C*_0_) vs. *t*.

A plot of −ln(*C/C*_0_) against (*t*) and slope of linear regression analysis is equal to the value of *K_app_*. The calculated results indicated that photocatalytic degradation at the reaction conditions follows pseudo-first-order kinetics (Figure 14). The values of *K_app_* were obtained directly from the regression analysis of the linear curve in the plot.

Table 1 summarizes the pseudo-first-order constants and correlation coefficients, and the results show that the photodegradation process follows a pseudo-first-order reaction. As shown in the Table 1, the reaction rate constant of Y_2_O_3_/TiO_2_-loaded polyester fabric is about four times higher than that of polyester, exhibiting good degradation activities. 

### 3.7. Self-Cleaning Performance Analysis

**Figure 15:**
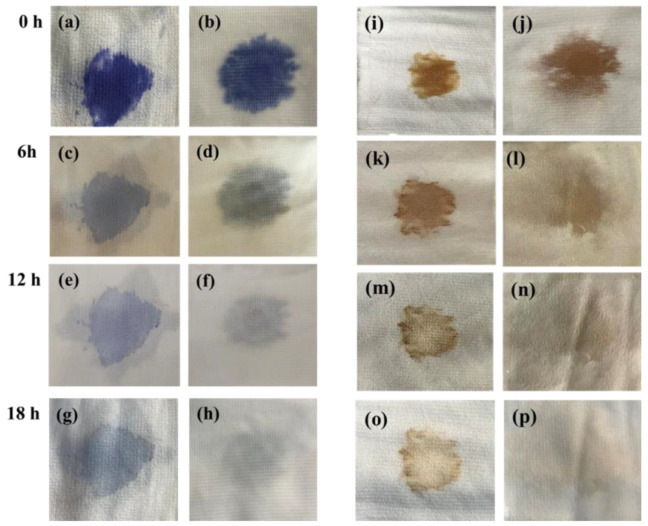
The self-cleaning performance of the Y_2_O_3_/TiO_2_-loaded polyester fabric to methyl blue and soy sauce: (**a**,**c**,**e**,**g**) are polyester fabrics with methyl blue; (**b**,**d**,**f**,**h**) are the polyester fabric with methyl blue and loaded with Y_2_O_3_/TiO_2_; (**i**,**k**,**m**,**o**) are the polyester fabric with soy sauce; (**j**,**l**,**n**,**p**) are Y_2_O_3_/TiO_2_ loaded polyester fabric dripped with soy sauce.

To test the self-cleaning performance of Y_2_O_3_/TiO_2_-loaded polyester fabric under simulated visible light, 0.1 mL of methyl blue solution and soy sauce were dropped on the polyester fabric and Y_2_O_3_/TiO_2_-loaded polyester fabric and then irradiated under a xenon lamp for 18 h. Sample images were taken every 6 h, and the results are shown in Figure 15. Figure 15a,c,e,g represent the polyester fabric with methyl blue solution. It can be found that the blue mark of the polyester fabric dyed with methyl blue basically remains after 6 h of xenon lamp irradiation. Figure 15b,d,f,h show the Y_2_O_3_/TiO_2_-loaded polyester fabric with methyl blue solution. It can be seen that the methyl blue was degraded significantly after irradiation for 18 h with the xenon lamp. Figure 15i,k,m,o show the polyester fabric with the applied soy sauce. Although the soy sauce was degraded after 12 h of irradiation, there were no obvious changes in color, and there still existed a large stain on the surface of the polyester fabric after 18 h of irradiation, indicating a low degradation rate for soy sauce on the surface of the polyester fabric. As shown in Figure 15j,l,n,p, the soy sauce on the Y_2_O_3_/TiO_2_-loaded polyester fabric began to degrade significantly after 6 h of light exposure and was completely decomposed after 18 h of xenon lamp irradiation. The reason for this is that the Y_2_O_3_/TiO_2_ will produce electron–hole pairs when irradiated by photons which have greater energy than its band gap energy [51], and the holes in the valence band can react with H^+^ and ⋅OH generated from adsorbed water, and the electrons can reduce oxygen to produce O^2−^. These active functional groups such as ⋅OH and O^2−^ could oxidize the organic compounds in methyl blue and soy sauce until they were completely mineralized [52]. 

## 4. Conclusions

Our Y_2_O_3_/TiO_2_ photocatalyst was prepared and treated with 3-aminopropyltrimethoxysilane (APTMS), and then the Y_2_O_3_/TiO_2_-loaded polyester fabric was successfully prepared via covalent bonding between Y_2_O_3_/TiO_2_ and the polyester fabric by the formation of an amide bond. 

The TiO_2_ in the Y_2_O_3_/TiO_2_ composite catalyst (anatase type) was uniformly distributed on the surface of the polyester fabric. The diffraction peaks obtained from the Y_2_O_3_/TiO_2_-loaded polyester fabric were in good agreement with those from Y_2_O_3_/TiO_2_ powder.

Furthermore, compared to polyester fabrics, the Y_2_O_3_/TiO_2_-loaded polyester fabrics had better photocatalytic performance under simulated sunlight. After 120 min of light exposure, the degradation rate of methyl orange reached 83% and when compared to the polyester fabric with the same drop of methyl blue solution and soy sauce, the Y_2_O_3_/TiO_2_-loaded polyester fabric had superior photocatalytic self-cleaning properties.

## Figures and Tables

**Table 1:** Pseudo-first-order rate constants of photocatalytic degradation of methyl orange.

Sample	Kapp (min^−1^)	R^2^
MO	0.0675	0.98398
polyester fabric	0.12725	0.99273
Y_2_O_3_/TiO_2_-loaded polyester fabric	0.47846	0.98363

## Data Availability

The data presented in this study are available on request from the corresponding author.
